# Maternal Analgesic Exposure and Fetal Ductal Constriction: A Prospective Cohort Study in Late Pregnancy

**DOI:** 10.1002/bdr2.70039

**Published:** 2026-02-27

**Authors:** Isaura Elaine Gonçalves Moreira Rocha, Andresa Carvalho Nobre, Beatriz Gonçalves Rocha, Paulo Henrique Benevides Siqueira, Estelita Lima Cândido, Simone Cristina Soares Brandão

**Affiliations:** ^1^ Federal University of Pernambuco (UFPE) Recife Brazil; ^2^ Federal University of Cariri (UFCA) Barbalha Brazil

**Keywords:** acetaminophen, drug safety, fetal ductus arteriosus, fetal hemodynamics, metamizole

## Abstract

**Objective:**

To evaluate the association between maternal analgesic exposure in late pregnancy and fetal ductal and pulmonary hemodynamics using serial echocardiographic assessment, and to explore potential differences between metamizole and acetaminophen.

**Methods:**

In this prospective cohort study, 67 third‐trimester pregnancies were evaluated: 47 exposed to analgesics (27 metamizole and 20 acetaminophen) and 20 unexposed controls. Two standardized fetal echocardiograms were performed: during exposure (T1) and after a 5–7 day drug‐free interval (T2). Ductal Doppler parameters, including systolic velocity, diastolic velocity, and pulsatility index (PI), were used to define ductal constriction based on established criteria (PI < 1.9 and/or increased velocities). Right‐heart and pulmonary hemodynamic parameters, including mean pulmonary artery pressure (MPAP) and acceleration time/ejection time ratio (AT/ET), were also assessed.

**Results:**

In the primary pooled analysis, ductal constriction occurred in 38.3% (18/47) of exposed fetuses and in 0% (0/20) of controls (*p* = 0.00065). When stratified by exposure, constriction was observed in 52% (14/27) of metamizole‐exposed fetuses and in 20% (4/20) of acetaminophen‐exposed fetuses. In multivariable analysis, metamizole use (OR 2.05; 95% CI 1.28–3.28), exposure within 48 h (OR 1.96; 95% CI 1.12–3.44), and dose > 1 g (OR 2.64; 95% CI 1.31–5.32) were independently associated with ductal constriction. Within the metamizole group, higher doses were associated with a greater proportion of constriction, although subgroup size limited statistical precision. After metamizole withdrawal, PI increased significantly (1.86 ± 0.43 to 2.28 ± 0.41; *p* < 0.001), accompanied by reductions in systolic and diastolic velocities (*p* < 0.05). In the acetaminophen group, mild and reversible constriction was observed, with modest PI improvement at T2 (2.20 ± 0.44 to 2.40 ± 0.29; *p* = 0.040) and no major velocity changes. Neither exposure significantly altered MPAP or AT/ET.

**Conclusions:**

Maternal exposure to analgesics in late pregnancy was associated with fetal ductal constriction compared with unexposed controls. The association was stronger for metamizole and consistent with a dose‐related pattern. Acetaminophen was associated with mild, reversible ductal involvement in a subset of fetuses. Reversibility after drug withdrawal supports a functional, prostaglandin‐mediated mechanism. These findings have important implications for maternal analgesic use in late gestation and highlight the value of Doppler surveillance following exposure to prostaglandin‐modulating agents.

## 1 Introduction

The fetal ductus arteriosus (DA) is a vital vascular conduit connecting the main pulmonary artery to the descending aorta, diverting approximately 80%–90% of right ventricular output away from the pulmonary circulation toward the systemic circuit. Under physiological conditions, the vessel closes spontaneously after birth in response to increased oxygen tension and reduced prostaglandin activity. Premature constriction of the DA in utero, particularly in late gestation, is a clinically significant event that can lead to right ventricular overload, tricuspid regurgitation, and persistent pulmonary hypertension of the newborn (Ishida et al. 2017; Hernández‐Díaz et al. 2007; Martinho et al. 2020).

Since the 1980s, maternal use of non‐steroidal anti‐inflammatory drugs (NSAIDs) after 24 weeks' gestation has been recognized as the main cause of drug‐induced ductal constriction through inhibition of cyclooxygenase (COX) and suppression of prostaglandin synthesis (Momma and Takeuchi 1983; Moise 1993; Koren et al. 2006). Non‐opioid analgesics are among the most commonly used medications during pregnancy; more than 60% of women report using them at some stage, often without medical supervision (Zafeiri et al. 2021; Price and Collier 2017).

In several countries, particularly in Latin America, metamizole (dipyrone) remains widely available and frequently used during pregnancy. Metamizole is a pyrazolone‐derived prodrug whose active metabolites, 4‐methylaminoantipyrine and 4‐aminoantipyrine, exert analgesic and antipyretic effects primarily through inhibition of COX isoenzymes and subsequent suppression of prostaglandin synthesis. Although pharmacologically effective and inexpensive, metamizole is not approved for use in the United States and several European countries because of its association with rare but potentially life‐threatening agranulocytosis. Despite these regulatory restrictions, pharmacoepidemiological studies from Brazil and other Latin American regions consistently report metamizole as the most frequently consumed analgesic during pregnancy, either alone or in fixed‐dose combinations, followed by acetaminophen (Mengue et al. 2001; Fonseca et al. 2002; Dal Pizzol et al. 2009). Both agents are routinely used for pain and fever control and are widely accessible as over‐the‐counter medications, including in emergency care settings. This regulatory divergence underscores the need for robust fetal safety data in populations where the drug remains widely prescribed.

Acetaminophen is a non‐selective cyclooxygenase inhibitor that acts predominantly at the peroxidase site of the enzyme, leading to reduced prostaglandin synthesis under low peroxide conditions. Although its anti‐inflammatory activity is relatively weak, acetaminophen effectively suppresses central prostaglandin E_2_ production and can also decrease peripheral prostaglandin levels in specific physiological environments, a mechanism potentially capable of inducing ductal vasoconstriction (Allegaert et al. 2021; Ovalı 2020). This functional effect is well established in neonatology, where acetaminophen is increasingly used as a therapeutic agent for pharmacological closure of patent ductus arteriosus in preterm infants (Xi et al. 2022). Importantly, acetaminophen readily crosses the placenta by passive diffusion due to its low molecular weight and lipophilicity, achieving fetal concentrations comparable to maternal levels, while placental metabolism and transporter‐mediated efflux modulate fetal exposure (Conings et al. 2019).

Despite their widespread use, the fetal safety profiles of metamizole and acetaminophen remain incompletely defined, particularly in late gestation. Acetaminophen has long been considered safe at therapeutic doses, but recent data have questioned its innocuity during pregnancy (Vane and Botting 1998; Levy et al. 1995). Metamizole, although pharmacologically distinct from classical NSAIDs, also inhibits COX isoenzymes and modulates prostaglandin pathways (Brinkman et al. 2025; Weintraub and Mankuta 2006; Arruza et al. 2008). Case reports and small observational series describing fetal ductal constriction temporally associated with maternal exposure to either drug have been reported, but causal evidence remains limited and has not been confirmed by prospective studies (Allegaert et al. 2019; Schierz et al. 2018; Dathe et al. 2019; Lopes et al. 2016). These uncertainties are clinically relevant given the widespread use of both drugs and the increasing ductal sensitivity to prostaglandin withdrawal in the third trimester (Moise 1993; Curvello et al. 2025).

Therefore, this prospective cohort study aimed to evaluate the association between maternal analgesic exposure during the third trimester and fetal ductal and pulmonary hemodynamics using serial echocardiographic assessment, and to explore potential differences between metamizole and acetaminophen.

## 2 Methods

### 2.1 Study Population

This prospective cohort study was conducted over an 18‐month period between August 2023 and April 2025 at a tertiary fetal cardiology unit in northeastern Brazil. Third‐trimester pregnant women were eligible for inclusion, with gestational ages ranging from 27 to 37 weeks at the time of the first fetal echocardiographic assessment. Inclusion criteria were singleton gestations with normal fetal structural cardiac anatomy on screening ultrasound and confirmed maternal exposure to metamizole or acetaminophen within 72 h prior to the first fetal echocardiogram (T1) (Moise 1993; Lopes et al. 2016).

Exclusion criteria included fetal structural heart disease; morphologic abnormalities of the ductus arteriosus (e.g., S‐shaped configuration, marked tortuosity, or angulated trajectory that could interfere with Doppler insonation); maternal infection; exposure to NSAIDs drugs or corticosteroids; maternal comorbidities such as chronic hypertension or diabetes mellitus; and recent or regular maternal intake of polyphenol‐rich foods and beverages known to induce fetal ductal constriction, including coffee, black or green tea, yerba mate, grape‐derived products, and dark chocolate, in accordance with published clinical recommendations (Zielinsky et al. 2012).

**FIGURE 1:**
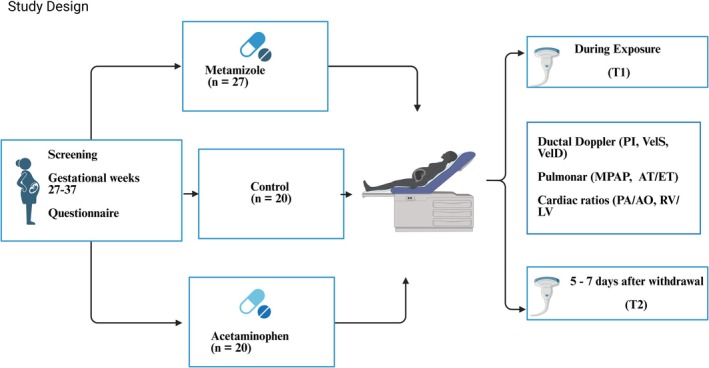
Study design illustrating the three study groups (metamizole, acetaminophen, and unexposed controls) and the two fetal echocardiographic assessments: T1, performed during maternal drug exposure, and T2, performed after a 5–7‐day drug‐free interval to assess reversibility of ductal and pulmonary hemodynamic changes.

The control group comprised healthy, unexposed third‐trimester pregnancies matched for maternal age and gestational age at examination (Allegaert et al. 2021). The overall study flow, including group allocation and timing of assessments (T1 and T2), is illustrated in Figure 1.

### 2.2 Exposure Definition

Exposure to metamizole and acetaminophen was classified based on maternal self‐reported use with prescription or medical record confirmation. Exposed pregnancies were eligible for inclusion if maternal drug intake had occurred within 72 h prior to the first fetal echocardiographic assessment (T1).

For temporal analyses, exposure timing was categorized as < 48 h versus ≥ 48 h (up to 72 h) before T1, based on standardized maternal reporting, allowing evaluation of proximity between drug intake and ductal hemodynamic changes (Dathe et al. 2019).

To assess reversibility of ductal involvement, all exposed participants underwent a second fetal echocardiographic assessment after a 5–7‐day drug‐free interval (washout period), following methodological approaches previously applied in studies evaluating ductal constriction resolution after discontinuation of the causal agent (Moise 1993; Dathe et al. 2019; Lopes et al. 2016).

Dosage and timing of exposure were recorded using a standardized maternal questionnaire. Drug doses were stratified as < 1 g or ≥ 1 g per intake, based on thresholds reported in prior clinical studies evaluating dose‐dependent ductal constriction risk (Curvello et al. 2025) and supported by recent pharmacodynamic data on metamizole (Brinkman et al. 2025). For all exposed pregnancies, the route of administration (oral or intravenous) was documented.

### 2.3 Sample Size Calculation

Sample size was estimated a priori to evaluate the occurrence of fetal ductal constriction among pregnancies exposed to prostaglandin‐modulating analgesics, based on previously reported constriction rates following pharmacological prostaglandin inhibition in late gestation. Prior studies have described frequencies ranging from approximately 15% to 50%, depending on drug class and gestational timing (Moise 1993; Koren et al. 2006; Zielinsky et al. 2024).

Assuming a conservative expected prevalence of 15%–20%, a two‐sided *α* of 0.05, and 90% power (*β* = 0.10), a minimum of 39 exposed pregnancies was required to estimate the prevalence of ductal constriction with acceptable precision and to detect a clinically meaningful occurrence above background rates. By the end of the recruitment period, 47 exposed cases had been included, exceeding the minimum required sample size.

The sample size calculation was based on the exposed cohort as a whole and was not designed to detect differences between individual drug groups. Comparisons between metamizole and acetaminophen were therefore prespecified as exploratory analyses.

An unexposed control group of third‐trimester pregnancies was included for comparative context. Participant recruitment followed a consecutive, non‐probabilistic sampling approach, including all eligible cases presenting during the study period.

### 2.4 Fetal Echocardiographic Protocol

Fetal echocardiography was performed using a Philips Affiniti 70 ultrasound system (Philips Medical Systems, Bothell, WA, USA) equipped with a 3–5 MHz convex transducer and pulsed‐wave Doppler. Doppler insonation angles were maintained below 20° whenever feasible to minimize velocity measurement error.

The ductus arteriosus (DA) was assessed primarily in the longitudinal ductal arch view and, when necessary, in the transverse three‐vessel‐and‐trachea (3VT) view to optimize Doppler alignment (< 30°) (Moon‐Grady et al. 2023). Peak systolic velocity (VelS), end‐diastolic velocity (VelD), and pulsatility index (PI = [Vmax − Vmin]/Vmean) were obtained following established Doppler acquisition standards (Moon‐Grady et al. 2023; Huhta et al. 1987).

Fetal ductal constriction was diagnosed in the presence of turbulent ductal flow associated with reduced PI (< 1.9) and elevated velocities (VelS ≥ 1.4 m/s and/or VelD ≥ 0.30 m/s) (Lopes et al. 2016; Moon‐Grady et al. 2023; Tulzer et al. 1991). Hemodynamic severity was classified as mild (isolated Doppler abnormalities), moderate (evidence of mild right‐heart overload), or severe (marked right‐heart dilation, significant tricuspid regurgitation, or fetal hydrops) (Tulzer et al. 1991; Genoves et al. 2015).

Additional echocardiographic parameters included right‐to‐left ventricular diameter ratio (RV/LV), pulmonary artery‐to‐aorta diameter ratio (PA/Ao), interventricular septal curvature, and qualitative assessment of tricuspid regurgitation to evaluate right ventricular strain (Rychik et al. 2023). Pulmonary circulation was further assessed by estimating mean pulmonary artery pressure (MPAP) using the Dabestani method (Dabestani et al. 1987) and calculating the acceleration‐to‐ejection time ratio (AT/ET) from pulmonary trunk Doppler flow profiles (Sosa‐Olavarría et al. 2019; Khoury et al. 2020).

All examinations were performed and analyzed by a single experienced fetal cardiologist who was blinded to maternal clinical characteristics and exposure status during image interpretation.

### 2.5 Outcomes

The primary outcome was the presence and hemodynamic severity of fetal ductal constriction, classified as mild, moderate, or severe according to predefined Doppler‐based criteria incorporating flow acceleration, reduction in pulsatility index (PI), and right‐heart hemodynamic repercussion (Lopes et al. 2016; Huhta et al. 1987; Tulzer et al. 1991). The primary inference was based on the pooled comparison between analgesic‐exposed pregnancies and unexposed controls.

Secondary outcomes included (i) exploratory drug‐stratified comparisons (metamizole vs. acetaminophen) in constriction frequency and severity; (ii) changes in pulmonary hemodynamics—mean pulmonary artery pressure (MPAP) and the acceleration‐to‐ejection time ratio (AT/ET)—between the initial assessment during drug exposure (T1) and follow‐up after drug discontinuation (T2) (Dabestani et al. 1987; Sosa‐Olavarría et al. 2019); and (iii) associations of gestational age, drug dose, and exposure timing (< 48 h vs. ≥ 48 h) with ductal Doppler parameters (VelS, VelD, and PI).

Reversibility was systematically evaluated at T2 and categorized as: Complete normalization, defined as resolution of ductal constriction with return to non‐constrictive Doppler criteria; Partial improvement, defined as a reduction in hemodynamic severity grade (e.g., moderate to mild) while persistent constriction criteria were still present.

### 2.6 Statistical Analysis

Analyses were performed using JASP v0.18.1 (University of Amsterdam, The Netherlands). Continuous variables were assessed for normality using the Shapiro–Wilk test and for equality of variances using Levene's test. Between‐group comparisons were conducted using one‐way ANOVA or Kruskal–Wallis tests, as appropriate. Within‐subject changes between T1 and T2 and group versus time interactions were evaluated using repeated‐measures ANOVA (or the non‐parametric equivalent when applicable).

Categorical variables were compared using *χ* ^2^ or Fisher's exact tests. Associations with ductal constriction were expressed as relative risks (RR) with 95% confidence intervals (CI) for bivariate analyses. Proportions were reported with exact binomial (Clopper–Pearson) 95% confidence intervals, and absolute risk differences were calculated with 95% confidence intervals using the Newcombe (Wilson) method. Correlations among gestational age, exposure timing (< 48 h vs. ≥ 48 h), dose, and ductal Doppler parameters were assessed using Spearman's rank correlation, and results were summarized in a correlation heatmap.

Multivariable logistic regression was used to identify independent predictors of ductal constriction, including drug group (metamizole vs. acetaminophen), dose category (≥ 1 g), exposure timing (< 48 h), and gestational age. Results are presented as adjusted odds ratios (OR) with 95% CI. Statistical significance was defined as *p* < 0.05.

## 3 Results

During the study period, 1.801 fetal echocardiograms were performed. Sixty‐seven third‐trimester pregnancies met the inclusion criteria: 27 exposed to metamizole, 20 to acetaminophen, and 20 unexposed controls. Maternal and gestational characteristics were comparable across groups. Headache and low back pain were the most common indications for analgesic use (Table 1).

**TABLE 1:** Maternal and gestational characteristics according to study group.

Variable	Dipyrone group (*n* = 27)	Paracetamol group (*n* = 20)	Control group (*n* = 20)
Maternal age (years), mean ± SD	31.3 ± 6.2	30.7 ± 5.9	31.2 ± 4.4
Gestational age (weeks), median (IQR)	29 (28–33.5)	29 (27–31.3)	28 (27.3–29.0)
Reported symptoms, *n* (%)			
Headache	11 (40.7)	12 (60.0)	—
Low back pain	8 (29.6)	2 (10.0)	—
Urinary tract infection	2 (7.4)	0 (0)	—
Abdominal pain	1 (3.7)	3 (15.0)	—
Toothache	0 (0)	1 (5.0)	—
Muscle pain	3 (11.1)	1 (5.0)	—
Arthralgia	1 (3.7)	1 (5.0)	—
Analgesic exposure, *n* (%)			
Dose > 1 g	17 (62.9)	5 (25.0)	—
Intravenous administration	5 (18)	—	
Use within 48 h before exam	18 (66.6)	17 (85.0)	—

*Note:* Data are presented as mean ± SD or median (IQR), as appropriate. g: gram; h: hour; IQR: interquartile range; *n*: absolute number; SD: standard deviation. This table summarizes descriptive baseline characteristics.

### 3.1 Incidence and Severity of Ductal Constriction

#### 3.1.1 Primary (Pooled) Analysis

Ductal constriction occurred in 38.3% (18/47) of exposed fetuses compared with 0% (0/20) of controls (Fisher's exact test, *p* = 0.00065). The estimated proportion among exposed pregnancies was 38.3% (95% CI 24.5–53.6), versus 0% in controls (95% CI 0–16.8), corresponding to an absolute risk difference of 38.3 percentage points (95% CI 9.7–52.6).

#### 3.1.2 Secondary (Drug‐Specific) Analyses

When stratified by exposure, constriction was observed in 51.9% (14/27) of metamizole‐exposed fetuses and in 20.0% (4/20) of acetaminophen‐exposed fetuses. All cases occurred exclusively among exposed pregnancies. In the metamizole group, eight cases were classified as mild and six as moderate; all acetaminophen‐related cases were mild.

### 3.2 Ductal Doppler Changes and Reversibility

In the metamizole group, repeated‐measures ANOVA demonstrated a significant group‐by‐time interaction. Between T1 (during exposure) and T2 (after 5–7 days without drug use), peak systolic velocity decreased from 1.26 ± 0.48 to 1.03 ± 0.39 m/s (*p* = 0.038), end‐diastolic velocity from 0.29 ± 0.24 to 0.18 ± 0.17 m/s (*p* = 0.045), and pulsatility index (PI) increased from 1.86 ± 0.43 to 2.28 ± 0.41 (*p* < 0.001).

**FIGURE 2:**
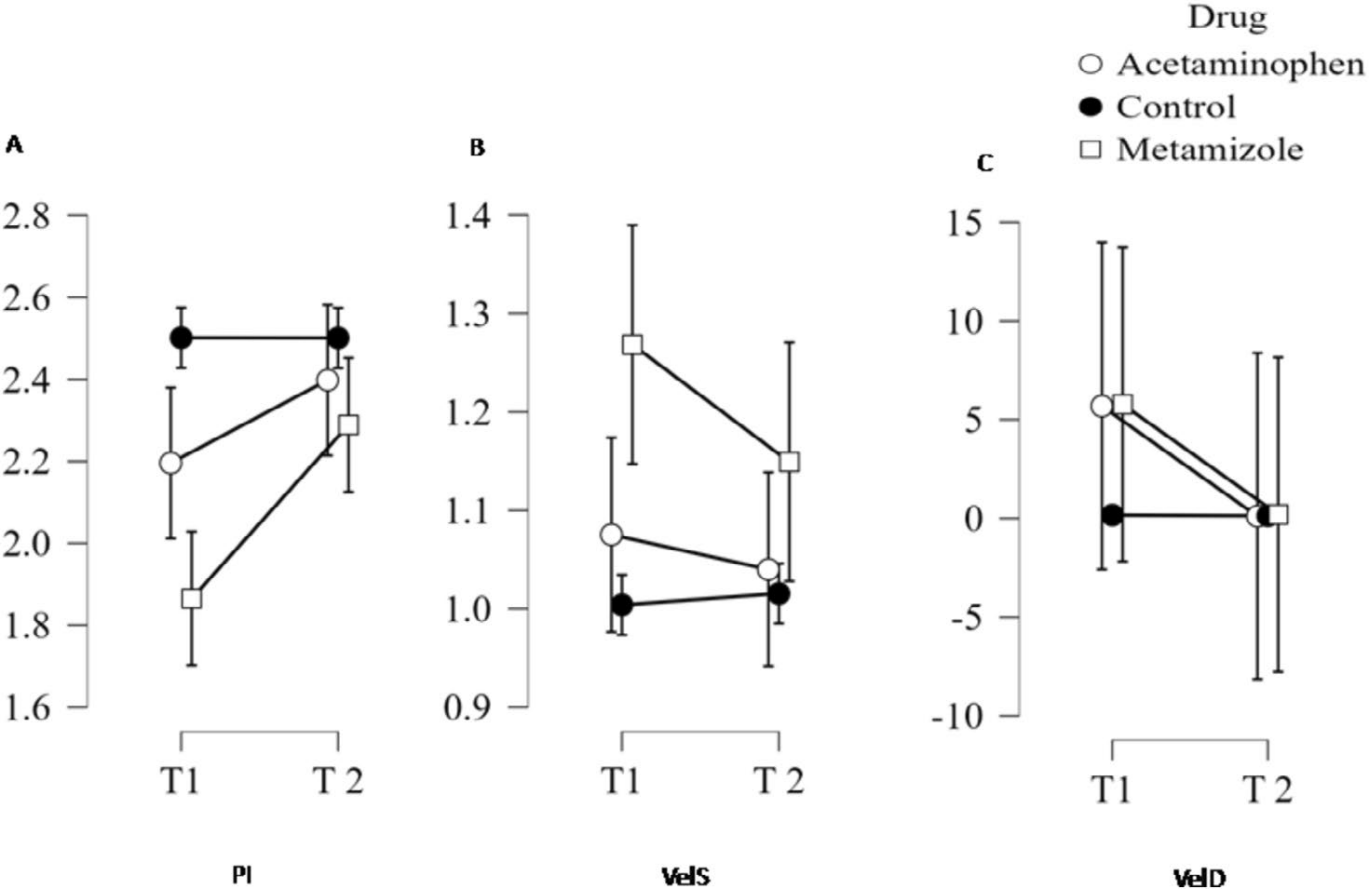
(A–C) Repeated‐measures analysis of fetal ductal Doppler parameters in pregnancies exposed to metamizole, acetaminophen, and unexposed controls. (A) Ductal pulsatility index (PI), (B) peak systolic velocity (VelS), and (C) end‐diastolic velocity (VelD) measured at T1 (during exposure) and T2 (after 5–7 days of drug withdrawal). In the metamizole group, PI increased and VelS and VelD decreased significantly after withdrawal (all *p* < 0.05). In the acetaminophen group, a mild but significant increase in PI was observed (*p* = 0.040), without significant changes in flow velocities. No significant changes were observed in the control group. Data are presented as mean ± standard deviation (SD). PI, pulsatility index; VelD, end‐diastolic velocity; VelS, systolic velocity.

In the acetaminophen group, no significant group‐by‐time interaction was detected. A modest increase in PI was observed after drug withdrawal (2.20 ± 0.44 to 2.40 ± 0.29; *p* = 0.040), while peak systolic and end‐diastolic velocities remained unchanged (both *p* > 0.05). All Doppler abnormalities resolved within 5–7 days. Serial Doppler findings are illustrated in Figure 2.

### 3.3 Comparison Between Constricted and Non‐Constricted Fetuses

**FIGURE 3:**
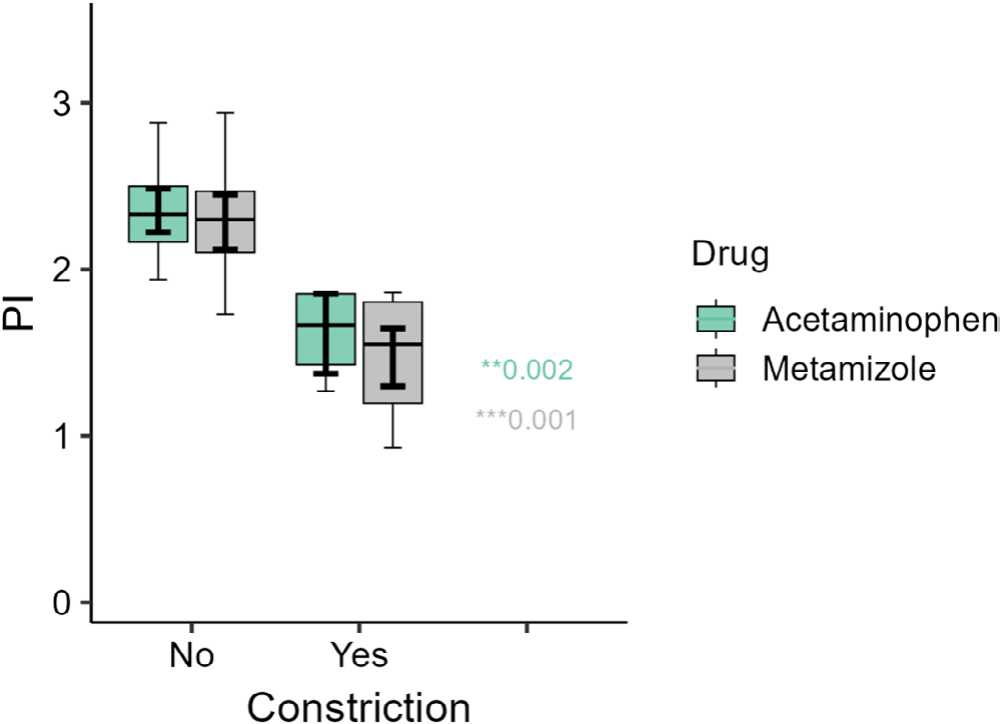
Comparison of ductal pulsatility index (PI) in fetuses exposed to metamizole or acetaminophen according to the presence or absence of ductal constriction. When constriction occurred, both drugs were associated with a similarly marked reduction in PI, indicating comparable hemodynamic severity despite different incidence rates. PI values were significantly lower in fetuses with ductal constriction (*p* = 0.001 for metamizole; *p* = 0.002 for acetaminophen). Data are presented as median and interquartile range (IQR).

Across exposed groups, fetuses with ductal constriction exhibited significantly lower PI values than those without constriction. In the metamizole group, PI was 1.48 ± 0.33 in constricted fetuses versus 2.28 ± 0.33 in non‐constricted fetuses (*p* < 0.001). In the acetaminophen group, PI values were 1.62 ± 0.29 versus 2.35 ± 0.28, respectively (*p* < 0.001) (Figure 3).

### 3.4 Association With Exposure, Dose, and Risk Estimates

The risk of ductal constriction was higher with metamizole than with acetaminophen (RR 2.59; 95% CI 1.00–6.70; Fisher's exact test *p* = 0.0359). This comparison should be interpreted as exploratory given subgroup sizes.

Within the metamizole group, ductal constriction was more frequent among fetuses exposed to doses ≥ 1 g (11/17, 64.7%) than among those exposed to doses < 1 g (3/10, 30.0%), corresponding to an unadjusted RR of 2.16 (95% CI 0.79–5.92; Fisher's exact *p* = 0.120). Although this suggests a dose–response pattern, subgroup size limits statistical precision.

In multivariable logistic regression, metamizole use (OR 2.05; 95% CI 1.28–3.28; *p* = 0.003), exposure within 48 h (OR 1.96; 95% CI 1.12–3.44; *p* = 0.018), and dose > 1 g (OR 2.64; 95% CI 1.31–5.32; *p* = 0.006) were independently associated with ductal constriction. Baseline ductal pulsatility index at T1 (PI1) was inversely associated with constriction (OR 0.34; 95% CI 0.18–0.65; *p* = 0.001). Acetaminophen use was not independently associated with constriction (OR 0.98; 95% CI 0.64–1.50; *p* = 0.920).

**FIGURE 4:**
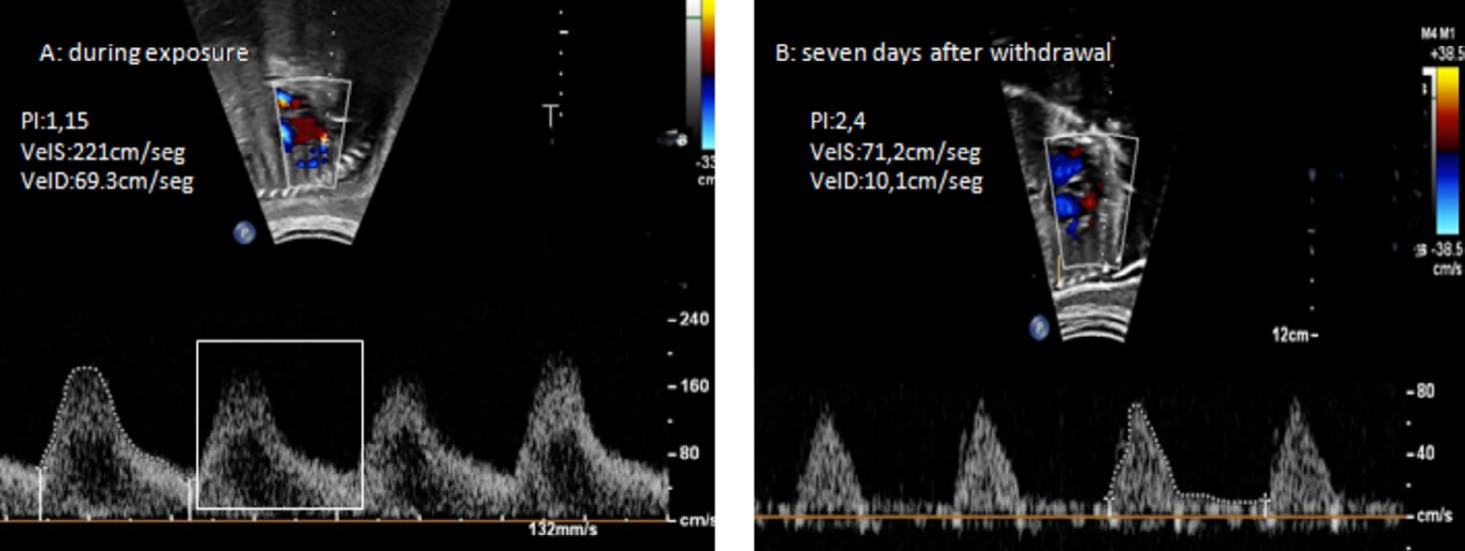
Reversible fetal ductal constriction during maternal metamizole exposure. (A) Marked ductal constriction characterized by turbulent flow, elevated end‐diastolic velocity (VelD; bracket), and reduced pulsatility index (PI = 1.15). (B) Normalization of ductal Doppler velocities and pulsatility index (PI = 2.40) 7 days after drug withdrawal. Images were obtained from the same fetus at 34 weeks' gestation using a longitudinal transabdominal sagittal view of the ductus arteriosus. A corresponding color Doppler video clip is available in the Supporting Information (Video S1).

After drug withdrawal, Doppler abnormalities improved in all 14 affected fetuses in the metamizole group: 10 (71.4%) showed complete normalization and 4 (28.6%) partial improvement. A representative case is shown in Figure 4.

In the acetaminophen group, four fetuses (20%) demonstrated mild ductal constriction, characterized by reduced PI values at T1 and normalization after drug cessation. Flow velocities did not change significantly (*p* > 0.05).

### 3.5 Pulmonary Hemodynamics and Secondary Findings

No significant differences were observed in pulmonary hemodynamic parameters, including mean pulmonary artery pressure (MPAP) and AT/ET ratio, across groups or between T1 and T2 (*p* > 0.05). In the metamizole group, septal bowing toward the left ventricle was documented in 3 fetuses (11.1%), and mild tricuspid regurgitation in 4 (14.8%), all among fetuses with ductal constriction. No similar findings were observed in the acetaminophen or control groups.

Exploratory correlation analyses are presented in Figure S1. The heatmap demonstrates inverse correlations between PI and ductal flow velocities, as well as an inverse correlation between MPAP and the AT/ET ratio. Gestational age showed positive correlations with systolic and diastolic ductal velocities and a mild inverse correlation with PI.

## 4 Discussion

This prospective cohort provides new evidence that maternal exposure to analgesics in late pregnancy is associated with fetal ductal constriction when compared with unexposed controls. In the primary pooled analysis, over one‐third of exposed fetuses met Doppler criteria for ductal constriction, whereas no cases were identified in the control group. When stratified by drug, the magnitude and pattern of effect differed substantially. Metamizole was more strongly associated with ductal constriction and demonstrated a pattern consistent with dose–response, whereas acetaminophen was associated with a measurable proportion of mild constriction (20%), occurring even at relatively low maternal doses and short exposure durations. The serial Doppler design strengthens the temporal association between exposure and hemodynamic alterations and confirms reversibility after drug withdrawal.

From a regulatory and international perspective, it is important to note that metamizole is not approved for use in the United States and several European countries due to concerns regarding rare but potentially severe maternal agranulocytosis (Brinkman et al. 2025; European Medicines Agency 2018). Nevertheless, it remains widely prescribed in Latin America and other regions because of its analgesic efficacy and low cost. This regulatory divergence underscores the relevance of generating fetal safety data in populations where metamizole continues to be used during pregnancy.

In Brazil and several Latin American countries, both metamizole and acetaminophen are widely available over the counter and are frequently used for self‐medication during pregnancy. In real‐world practice, these agents are often selected interchangeably for common symptoms such as headache or musculoskeletal pain. This pattern of exposure underscores the public health relevance of evaluating fetal hemodynamic effects in populations where analgesic use is common and may occur without direct medical supervision.

Both drugs share the ability to inhibit cyclooxygenase activity and reduce prostaglandin synthesis, a mechanism known to influence ductal tone (Ovalı 2020; Vane and Botting 1998). In late gestation, increasing ductal smooth muscle sensitivity to prostaglandin withdrawal predisposes the vessel to functional constriction (Momma and Takeuchi 1983). The observed reductions in pulsatility index and increases in ductal velocities during exposure, followed by rapid normalization after drug discontinuation, are consistent with this mechanism and mirror experimental and clinical models of prostaglandin‐dependent ductal constriction (Moise 1993; Zielinsky et al. 2012).

Our results extend the observations of Moise et al. (Moise 1993) regarding prostaglandin‐inhibition‐induced constriction and support growing evidence that non‐NSAID analgesics may also affect ductal tone. The incidence of constriction among metamizole‐exposed fetuses (approximately 52%) was similar to rates reported for indomethacin, reinforcing a prostaglandin‐mediated mechanism.

In contrast, acetaminophen exposure was associated with mild ductal involvement in a subset of cases. Although this proportion (20%) suggests that acetaminophen may not be entirely benign in late gestation, the subgroup size limits precision, and these findings should be interpreted cautiously. The prospective design and serial Doppler assessment in our study likely increased detection sensitivity, supporting the hypothesis that acetaminophen may exert subtle yet measurable effects on ductal tone in susceptible fetuses (Allegaert et al. 2019; Schierz et al. 2018; Dathe et al. 2019).

Importantly, acetaminophen is widely used in neonatology as a standard pharmacological therapy for closure of patent ductus arteriosus in preterm infants, providing strong biological plausibility for ductal sensitivity to this drug (Allegaert et al. 2021; Xi et al. 2022). Emerging reports of intrauterine ductal constriction following maternal acetaminophen exposure further support this concern (Hauben et al. 2021; Becquet et al. 2018). In this context, the modest but reversible Doppler changes observed in our study suggest that acetaminophen‐related ductal effects may be clinically relevant in selected cases.

Exploratory correlation analyses provided additional physiological insight, particularly regarding gestational age (GA). In the metamizole group, GA showed positive correlations with systolic and diastolic ductal velocities and an inverse correlation with PI‐patterns consistent with normal maturation, in which increasing muscular tone and declining prostaglandin sensitivity physiologically elevate flow velocities. The greater magnitude of these associations among exposed fetuses suggests that metamizole may potentiate the late‐gestation rise in ductal contractility. Lower PI also correlated with higher PA/Ao and RV/LV ratios, reinforcing the link between reduced ductal compliance and increased right ventricular afterload. The inverse relationship between MPAP and AT/ET mirrored established pulmonary hemodynamics, indicating that even transient constriction may modulate fetal pulmonary vascular resistance.

Although no significant differences were observed in pulmonary artery pressure or AT/ET ratio, the presence of mild tricuspid regurgitation and septal bowing in some metamizole‐exposed fetuses demonstrates that short‐lived constriction can cause measurable hemodynamic effects. The absence of persistent pulmonary hypertension suggests that these alterations were functional and reversible.

Differences between drugs likely reflect variations in COX‐inhibitory potency and placental transfer (Price and Collier 2017). Metamizole's active metabolite, 4‐methylaminoantipyrine, crosses the placenta efficiently and accumulates in fetal plasma, whereas acetaminophen shows a more balanced maternal‐fetal distribution and faster clearance (Conings et al. 2019). These pharmacokinetic characteristics are consistent with the more frequent and more pronounced constrictive response observed with metamizole.

Although we adopted a pulsatility index threshold of ≤ 1.9 in accordance with classical Doppler definitions (Huhta et al. 1987; Mielke and Benda 2000; Tulzer et al. 1991), more recent reference curves propose a higher cutoff (≤ 2.2), corresponding to the fifth percentile of gestational age‐adjusted normal values (Zielinsky et al. 2024). Application of this updated threshold would likely have identified additional affected fetuses in our cohort and therefore would be expected to reinforce, rather than weaken, the observed associations.

Several methodological considerations merit discussion. The sample size was calculated to detect ductal constriction among exposed pregnancies overall; comparisons between metamizole and acetaminophen were exploratory and not based on assumed equivalence of effect. The gestational age range (27–37 weeks) represents a potential limitation, as the ductus arteriosus becomes progressively more sensitive to prostaglandin withdrawal closer to term (Momma and Takeuchi 1983). Given that mean gestational age in our cohort was in the earlier third trimester, the magnitude of drug‐related effects may have been underestimated.

Potential confounding by dietary polyphenol intake was minimized by explicitly excluding pregnancies with recent or regular consumption of polyphenol‐rich foods known to induce ductal constriction, in accordance with current clinical recommendations (Zielinsky et al. 2012). This strengthens the attribution of observed effects to pharmacological exposure.

Strengths of this study include its prospective design, standardized echocardiographic protocol, and use of serial assessments to evaluate reversibility. The sample exceeded the a priori estimated requirement, ensuring adequate power for the primary pooled comparison between exposed and unexposed pregnancies. Limitations include the modest subgroup sizes, absence of maternal or fetal drug‐level quantification, and incomplete blinding at the time of examination, although retrospective anonymized review minimized classification bias. As this was an observational study based on maternal self‐reported use, both dose and duration may have been underestimated. Notably, only five participants in the metamizole group received intravenous formulations, while most exposures were oral, a factor that may have limited peak fetal drug levels and attenuated acute constrictive effects. Because the cohort consisted of low‐risk pregnancies, generalizability to high‐risk populations or repeated high‐dose exposures may be limited.

### 4.1 Clinical Implications

Given the widespread availability and frequent use of over‐the‐counter analgesics during pregnancy, these findings underscore the importance of cautious prescribing in the third trimester. Metamizole was independently associated with fetal ductal constriction and should be carefully considered when evaluating analgesic options during pregnancy (Brinkman et al. 2025). Acetaminophen, although comparatively safer, was associated with mild ductal involvement in a subset of fetuses and should be used at the lowest effective dose and shortest duration in late gestation (American College of Obstetricians and Gynecologists 2021). Fetal echocardiography plays a key role in detecting early ductal constriction and guiding management, and repeat Doppler assessment may be considered following significant maternal exposure to prostaglandin‐inhibiting agents.

## 5 Conclusions

Maternal exposure to analgesics in late pregnancy was associated with fetal ductal constriction, with a markedly stronger and dose‐dependent effect observed for metamizole. Acetaminophen exposure was associated with mild and reversible ductal involvement in a subset of cases. The rapid normalization of Doppler findings after drug discontinuation supports a functional and reversible mechanism. As the first prospective cohort with systematic post‐discontinuation assessment, this study provides clinically relevant evidence to inform safer analgesic use and highlights the importance of fetal surveillance following exposure to prostaglandin‐modulating agents in the third trimester.

## Funding

The authors have nothing to report.

## Conflicts of Interest

The authors declare no conflicts of interest.

## Supporting information


**Video S1:** Color Doppler clip demonstrating turbulent ductal flow during maternal metamizole exposure. Bidimensional and color Doppler imaging of the fetal ductus arteriosus showing marked turbulence and aliasing at the narrowest ductal segment, consistent with functional ductal constriction. This video corresponds to the case illustrated in Figure 4A.

**Figure S1:** Spearman correlation heatmap for the metamizole group at T1. Strong and physiologically coherent associations were observed, including an inverse correlation between ductal pulsatility index (PI) and both systolic and end‐diastolic ductal velocities, as well as the expected negative correlation between mean pulmonary artery pressure (MPAP) and the acceleration‐to‐ejection time ratio (AT/ET). These findings highlight an integrated hemodynamic response involving ductal compliance and pulmonary vascular load. Correlation matrices for the acetaminophen group showed weak and inconsistent associations and are therefore not shown.

## Data Availability

The data that support the findings of this study are available on request from the corresponding author. The data are not publicly available due to privacy or ethical restrictions.
