# Supplementary figures and images for: Maternal Analgesic Exposure and Fetal Ductal Constriction: A Prospective Cohort Study in Late Pregnancy
Source: Birth Defects Res. 2026 Feb 27;118(3):e70039. doi: 10.1002/bdr2.70039 (PMC12948653; doi:10.1002/bdr2.70039)

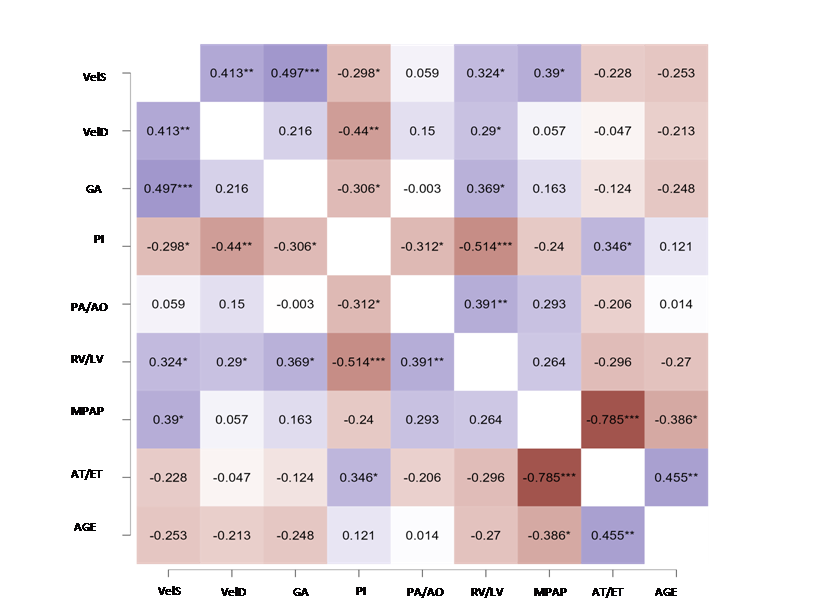

Supplement: Supplementary file 2 — Figure S1: Spearman correlation heatmap for the metamizole group at T1. Strong and physiologically coherent associations were observed, including an inverse correlation between ductal pulsatility index (PI) and both systolic and end‐diastolic ductal velocities, as well as the expected negative correlation between mean pulmonary artery pressure (MPAP) and the acceleration‐to‐ejection time ratio (AT/ET). These findings highlight an integrated hemodynamic response involving ductal compliance and pulmonary vascular load. Correlation matrices for the acetaminophen group showed weak and inconsistent associations and are therefore not shown. [file BDR2-118-e70039-s002.png]
